# Status-dependent metabolic effects of social interactions in a group-living fish

**DOI:** 10.1098/rsbl.2024.0056

**Published:** 2024-07-24

**Authors:** André Morin, Brett M. Culbert, Hossein Mehdi, Sigal Balshine, Andy J. Turko

**Affiliations:** ^1^ Department of Psychology, Neuroscience & Behaviour, McMaster University, 1280 Main Street West, Hamilton, Ontario, Canada; ^2^ School of Life and Environmental Sciences, Deakin University, 75 Pigdons Road, Geelong, Victoria, Australia; ^3^ Department of Integrative Biology, University of Guelph, 50 Stone Road East, Guelph, Ontario, Canada

**Keywords:** Cichlidae, metabolic recovery, respirometry, social buffering, social rank, stress

## Abstract

Social interactions can sometimes be a source of stress, but social companions can also ameliorate and buffer against stress. Stress and metabolism are closely linked, but the degree to which social companions modulate metabolic responses during stressful situations—and whether such effects differ depending on social rank—is poorly understood. To investigate this question, we studied *Neolamprologus pulcher*, a group-living cichlid fish endemic to Lake Tanganyika and measured the metabolic responses of dominant and subordinate individuals when they were either visible or concealed from one another. When individuals could see each other, subordinates had lower maximum metabolic rates and tended to take longer to recover following an exhaustive chase compared with dominants. In contrast, metabolic responses of dominants and subordinates did not differ when individuals could not see one another. These findings suggest that the presence of a dominant individual has negative metabolic consequences for subordinates, even in stable social groups with strong prosocial relationships.

## 1. Introduction

Social interactions can either increase or reduce physiological stress depending on the nature of the exchange [1–3]. Aggressive interactions generally cause stress (i.e. social subordination; [1]), whereas prosocial or affiliative interactions like allo-grooming can decrease stress (i.e. social buffering; [3,4]). Social buffering is known to influence several aspects of stress responses and recovery [3], but these effects have primarily been evaluated by measuring levels of glucocorticoid hormones (i.e. cortisol or corticosterone; [5]), particularly during interactions between pair-bonded mates or kin (e.g. parent–offspring; [4,6,7]). The potential metabolic benefits of prosocial exchanges, especially between non-kin group mates, have received much less attention despite marked fitness benefits of these relationships (e.g. enhanced lifespan and greater offspring survival; [8,9]). Furthermore, most studies of social buffering have focused on mammals, with far fewer investigations of other taxa such as fishes. A few previous studies have shown that social buffering can occur in non-territorial, shoaling species such as zebrafish (*Danio rerio*) [10,11], but the role of social buffering in fishes that live in more structured groups remains poorly understood. A complicating factor is that the hierarchical social structure observed in many groups means that larger, stronger individuals often become dominant and monopolize resources using aggression [12,13]. However, despite reduced access to food and shelter, subordinates generally still reap survivorship benefits by remaining in a group [14,15]. This is especially true when subordinates use prosocial behaviours to maintain strong ties with their group mates and thereby receive protection from larger, more dominant individuals during intergroup conflicts and predator attacks [16,17]. Considering the above trade-offs, we hypothesize that strong prosocial relationships may confer greater benefits to subordinates than dominants, which may be reflected by better energetic outcomes for subordinates.

Coping with a stressful social environment can impose metabolic disadvantages owing to—among other factors—chronically elevated levels of glucocorticoid hormones and the performance of energetically taxing social behaviours (e.g. aggression and/or submission [1,5]), both of which can directly affect standard metabolic rate and maximum metabolic capacity [18–21]. However, the potential beneficial effects of prosocial interactions among group mates on metabolic responses to stress are not well understood. Therefore, further characterization of how hormones, behaviour and the social environment (i.e. the presence or absence of group mates) collectively influence metabolic responses to stress would provide crucial insight into the complex interactions between these factors. There is some evidence hinting that the metabolic consequences of prosocial interactions between group mates may be substantial. For example, in a cooperatively breeding bird (chestnut-crowned babbler; *Pomatostomus ruficeps*) that lives in stable social groups, basal metabolic rates were 15% lower when individuals were held with at least three of their group mates [22]. However, metabolic rates reported in this study represent individual-adjusted data that were collected from groups of birds, and therefore do not indicate whether the presence of group mates differentially affected dominants versus subordinates. In dyads of juvenile damselfish (*Pomacentrus amboinensis*), dominant fish that were re-exposed to their subordinate counterpart 2 h after their initial interaction increased their metabolic rate by 60%, but metabolic rates of subordinates did not change when the dominant was present [23]. It remains unknown whether an individual’s social rank influences its metabolic response to stress in a species that forms long-lasting groups with strong prosocial relationships. To investigate this uncertainty, we measured metabolic rates (oxygen consumption) of the group-living African cichlid *N. pulcher*. This cooperatively breeding fish forms stable social groups consisting of a dominant breeding pair and anywhere from 1 to 20 subordinates organized in a linear dominance hierarchy [24]. Dominant fish often behave aggressively towards subordinates [24], but group mates also regularly exchange affiliative behaviours that promote group cohesion [25,26]. These affiliative relationships appear important for this fish species since the presence of group mates reduced circulating cortisol levels in subordinates following an acute stressor [27], which is indicative of social buffering.

Our objective was to assess whether the metabolic responses of dominants and subordinates are differentially affected by the presence of a group mate. We used intermittent flow respirometry to measure resting, routine and maximum metabolic rates (oxygen consumption) of paired dominant and subordinate female *N. pulcher* from the same social group. We focused on females because affiliative relationships are stronger in female versus male *N. pulcher* [28] and compared with males, females are more likely to assume a dominant position within their natal group [29,30]. Dominants and subordinates were placed in adjacent respirometry chambers where they could either visually interact (separated by transparent chamber walls) or not (separated by an opaque barrier). Metabolic rates were measured at rest and following an acute stressor (exhaustive chase) to determine the effect of social relationships on resting and routine metabolic rates, as well as metabolic responses to stress (maximum metabolic rate, aerobic scope and rates of metabolic recovery). If the presence of a social companion reduces physiological stress (i.e. if social buffering occurs), then fish that were allowed to visually interact should display greater metabolic benefits than isolated fish. This would be evidenced by visually interacting fish displaying reduced resting metabolism and faster recovery following exhaustion compared with isolated fish. Furthermore, since subordinates likely benefit more from the presence of social companions owing to increased protection, we also tested the prediction that the metabolic benefits of social interactions should be greater for subordinates versus dominants.

## 2. Methods

Experiments were conducted between February and October 2022 with laboratory-reared *N. pulcher*, which were descendants of wild-caught fish from Lake Tanganyika, Africa. Fish were held in 189 l aquaria containing a social group consisting of a dominant male–female breeding pair and 2–6 smaller subordinates of mixed sex [31]; these groups were established and subsequently allowed to stabilize for a minimum of one month before experimentation. Water temperature was held at 27°C (±1°C), and the photoperiod was light : dark for 13 : 11 h. Fish were fed 6 days per week with commercial cichlid flakes. Additional details on experimental animals and housing conditions are provided in the electronic supplementary materials.

**Figure 1.**
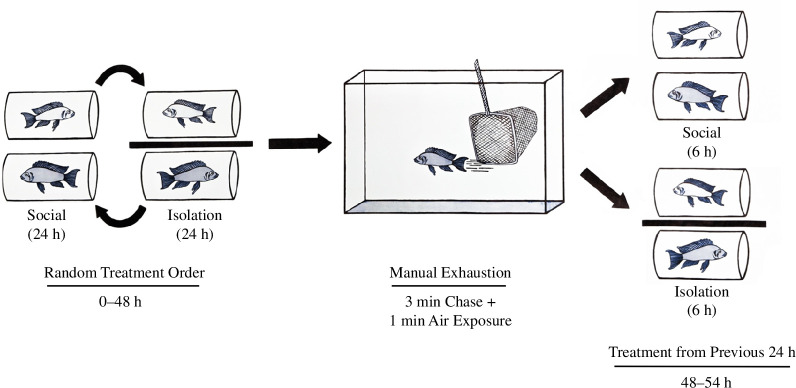
Overview of experimental design. Resting and routine metabolism of dominant and subordinate *N. pulcher* was measured during 0‒48 h. A group mate was visible for a 24 h period and concealed for the other 24 h period (order was randomized). After 48 h, fish were simultaneously chased in separate aquaria to exhaustion to elicit maximum metabolic rate. Following the chase, aerobic scope and measures of metabolic recovery were recorded between 48 and 54 h either in the presence or absence of a fish’s group mate (matching treatment from the previous 24 h). For illustrative purposes, dominants are slightly darker and larger than subordinates in the figure.

The dominant female and the largest subordinate female from each of the 16 social groups were placed in adjacent respirometry chambers for 48 h to measure resting and routine oxygen consumption rates (MO_₂_). This period was divided into a 24 h ‘social’ and a 24 h ‘isolation’ period which were conducted in a random order (figure 1). Transparent respirometry chambers were used to allow pairs to visually interact for the ‘social’ treatment, while an opaque barrier prevented visual interaction for the ‘isolation’ treatment [23]. Resting MO_₂_ [32] was calculated using the 10 lowest oxygen consumption measurements taken overnight. Routine MO_₂_, the average metabolic rate required for daily maintenance behaviours [32], was calculated using the five consecutive oxygen consumption periods which occurred 30 min after lights reached full intensity following the simulated sunrise. This window of time was used because fish were fully habituated after being in the respirometer for approximately 24 h and were manipulated shortly after this period (e.g. addition or removal of opaque barrier, measurement of maximum MO_₂_). To assess maximum MO_₂_, social pairs were simultaneously chased with a net for 3 min with each fish in separate aquaria followed by 1 min of air exposure [33]. Fish were then synchronously returned to their respective respirometry chambers, and oxygen uptake was measured for 6 h while fish recovered. This protocol was designed to measure maximum aerobic capacity of individual fish and does not reflect the ability of fish to modulate energy expenditure in response to stressors of varying magnitudes. We therefore interpret larger values of maximum metabolic rate as a higher capacity for aerobic performance. In these experiments, some pairs (*n* = 9) recovered while in visual contact with their group mate, while other pairs (*n* = 7) recovered in isolation (figure 1). Aerobic scope (AS), the capacity to increase aerobic metabolism, was calculated as the difference between maximum MO_₂_ and routine MO_₂_. To measure both short-term and long-term recoveries, we measured recovery time to 50% of AS (AS_50_) [23,34] and recovery time to within 10% of treatment-matched routine MO_₂_ [35], respectively. Finally, we calculated excess post-exercise oxygen consumption (EPOC) to determine the total amount of oxygen required for fish to recover to their routine MO_₂_ after exhaustion [36]. Measurement and flush periods were each 5 min long, resulting in the collection of approximately 320 oxygen consumption measurements for each individual fish per trial. Additional details are provided in the electronic supplementary materials.

Statistical analyses were performed using R (v. 4.3.1 [37]) and a significance level (*α*) of 0.05 was used for all tests. We used linear mixed models that included rank, visual condition, the interaction between rank and visual condition and log body mass as fixed effects. To account for repeated measurements of resting and routine metabolic rates (i.e. isolation and social), individual fish ID nested within pair ID (1|pairID/fishID) was initially included as a random effect in these models [38,39]. However, only pair ID (1|pairID) was included in the final model for routine MO_₂_ owing to issues with model singularity. Similarly, in other models (max MO_₂_, AS, time to AS_50_, time to 10% routine MO_₂_ and EPOC), pair ID (1|pairID) alone was included as a random effect to account for measurements from individuals that originated from the same social group. In the case of the time to 10% routine MO_₂_, including the random effect term violated the singularity assumption. As a result, we performed this analysis with and without the random effect term and the results were highly similar. For consistency with our other analyses, we present the results of the model that include the random effect term. All models were fitted with the ‘lme4’ package [40], while the ‘anova’ function from the ‘lmerTest’ package [41] was used to calculate *P* and *F*_DF_ values via type 3 tests and sum-zero contrasts. Tukey’s post hoc analysis was performed using the ‘emmeans’ package [42] when differences were detected. We also estimated effect sizes by calculating partial eta-squared (*η*_p_^2^) values using the ‘effectsize’ package [43]. Further explanation of data and statistical analysis can be found in the electronic supplementary materials.

## 3. Results

**Figure 2.**
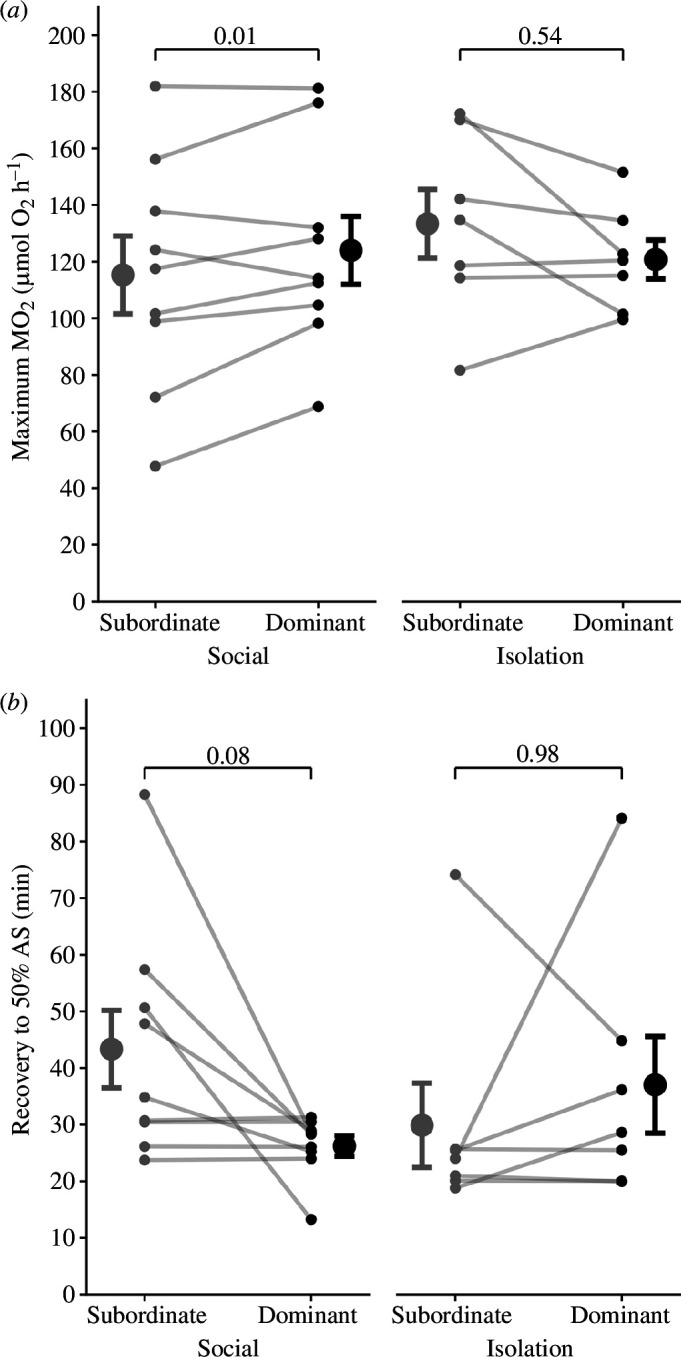
Maximum MO_2_ (*a*) and recovery time to 50% of aerobic scope (*b*) of subordinate and dominant *N. pulcher* that were allowed to visually interact (social) or prevented from interacting (isolation). Grey and black circles represent subordinates and dominants, respectively. Connecting lines indicate a dominant–subordinate pair from the same social group. Larger circles represent the mean ± standard error. Post hoc pairwise comparison *p*-values are shown above each plot.

When group mates could see one another directly before and after the chase, subordinate fish had 7% lower maximum metabolic rates than dominants (LMM: *F*_1,16_ = 5.46, *p* = 0.03; post hoc *p* = 0.01; figure 2*a*). Subordinates also had a slightly lower aerobic scope (by 4%), and this effect was observed regardless of whether a companion was visually available or not (*F*_1,23_ = 6.17, *p* = 0.02; table 1, electronic supplementary material, figure S3). The presence of a companion differentially affected how quickly dominants versus subordinates recovered to 50% of aerobic scope after experiencing the acute stressor (*F*_1,16_ = 6.84, *p* = 0.02; table 1). Subordinates took approx. 65% longer to recover to AS_50_ compared with dominants when fish could see their companion (post hoc *p* = 0.08; figure 2*b*), but there was no difference when fish were not in visual contact (post hoc *p* = 0.98). While we observed a tendency for EPOC to be influenced by a statistical interaction between social status and the presence of a companion (*F*_1,16_ = 4.44, *p* = 0.052; table 1), all post hoc differences were highly non-significant (*p* > 0.57; electronic supplementary material, figure S2*a*). No differences were detected for recovery to 10% of routine MO_₂_ (table 1, electronic supplementary material, figure S2*b*). Similarly, no differences in resting or routine metabolic rates were detected between dominant and subordinate fish, and these rates were also unaffected by the presence or absence of a companion (table 1, electronic supplementary material, figure S1).

**Table 1.** Summary statistics of statistical models evaluating metabolic and recovery traits of *N. pulcher*. Significant differences (*p* < 0.05) are indicated with bold font.

experimental stage	dependent variable	independent variable	*F*_DF_	*ƞ*_p_^2^	*P*
***metabolic rates***	resting MO_2_ (µmol O_2_ h^−1^)	status	1.03_(1,22)_	0.05	0.32
		treatment	0.01_(1,32)_	<0.001	0.98
		status × treatment	0.11_(1,32)_	0.003	0.74
		***body mass***	***6.35_(1,25)_***	***0.20***	***0.02***
	routine MO_2_ (µmol O_2_ h^−1^)	status	0.02_(1,64)_	<0.001	0.88
		treatment	1.38_(1,48)_	0.03	0.25
		status × treatment	1.29_(1,48)_	0.03	0.26
		***body mass***	***11.65_(1,61)_***	***0.16***	***0.001***
***response to acute stressor***	maximum MO_2_ (µmol O_2_ h^−1^)	***status***	***8.63_(1,18)_***	***0.32***	***0.01***
		treatment	0.30_(1,16)_	0.02	0.59
		***status × treatment***	***5.46_(1,16)_***	***0.26***	***0.03***
		***body mass***	***113.65_(1,19)_***	***0.86***	***<0.001***
	aerobic scope (AS; µmol O_2_ h^−1^)	***status***	***6.17_(1,23)_***	***0.21***	***0.02***
		treatment	<0.001_(1,16)_	<0.001	0.99
		status × treatment	0.01_(1,15)_	<0.001	0.91
		***body mass***	***50.04_(1,27)_***	***0.65***	***<0.001***
***recovery from acute stressor***	time to 50% of aerobic scope (AS_50_; min)	status	1.91_(1,29)_	0.06	0.18
		treatment	0.41_(1,16)_	0.02	0.53
		***status × treatment***	***6.84_(1,16)_***	***0.30***	***0.02***
		body mass	0.82_(1,27)_	0.03	0.37
	time to 10% of routine MO_2_ (min)	status	0.80_(1,27)_	0.03	0.38
		treatment	1.39_(1,27)_	0.05	0.25
		status × treatment	1.18_(1,27)_	0.04	0.29
		body mass	0.07_(1,27)_	0.003	0.79
	excess post-exercise oxygen consumption (EPOC; µmol O_2_)	status	0.23_(1,26)_	0.009	0.63
		treatment	0.14_(1,16)_	0.009	0.71
		status × treatment	4.44_(1,16)_	0.22	0.052
		***body mass***	***9.78_(1,31)_***	***0.24***	***0.004***

## 4. Discussion

We found no evidence that the presence of a familiar group mate buffered metabolic responses to stress. In fact, subordinates took longer to recover from an acute exhaustive stressor and had a lower maximum MO_₂_ when a familiar dominant fish was present. Thus, the ability of subordinates to mount and recover from a stress response was impaired in the presence of a dominant fish. In social groups, subordinate *N. pulcher* perform submissive behaviours [44] and provide allocare for the offspring of the dominant breeding pair [45]—while also suppressing their own reproductive output [46]—as these actions reduce aggression received from dominants. The performance of these behaviours (e.g. submissive displays), is energetically costly for subordinates [19] and might necessitate metabolic adjustments that compromise the ability of subordinates to appropriately regulate their metabolism in response to acute stress (e.g. reduced maximum MO_₂_ and longer recovery times). Our results may also reflect social transmission of stress between group mates, wherein individuals display a stronger and/or prolonged stress response after interacting with a stressed conspecific [2]. Indeed, current evidence indicates that the physiological state of group mates appears to be a major driver of stress responses in *N. pulcher*. For example, Culbert *et al*. [27] reported behavioural and physiological evidence consistent with social buffering when a stressed subordinate recovered with its non-stressed group mates, but we observed no evidence of social buffering when all fish were similarly stressed. Taken together, these results indicate that the occurrence of social buffering is highly context-specific in *N. pulcher* as has been reported in other species [47]. Further work is needed to understand why some social contexts result in social buffering while others do not. Specifically, when designing future studies, it will be important to account for stressed versus unstressed conspecifics, individual companion versus group stimulus and the relative rank of the conspecifics.

There are several possible explanations for the reduced maximum MO_₂_ and longer recovery times that we measured in subordinate fish. One possibility is that subordinates had smaller energy reserves than dominants, limiting energy mobilization. However, subordinate *N. pulcher* usually maintain greater energy reserves than dominants [48,49], making this hypothesis unlikely. A second possibility is that subordinates had a reduced capacity to mobilize their energy reserves in the presence of a dominant fish. Energy mobilization in response to stress primarily occurs via the combined actions of glucocorticoids and catecholamines [50]. It is unlikely that our observed results are mediated by differences in glucocorticoid responses. While resting cortisol levels are sometimes higher in subordinate *N. pulcher* [51–53], cortisol responses following acute stressors are identical between ranks [52]. Furthermore, elevated cortisol levels typically increase maximum MO_₂_ [18,21], opposite to what we observed for subordinates. Less is known about how the social environment affects the regulation of catecholamines, but previous studies suggest that subordinates may have elevated basal activation of catecholamine synthesis [54,55]. This tonic activation could impair the ability of subordinates to increase systemic catecholamine levels in response to acute stressors because of depleted catecholamine stores and/or desensitization of release mechanisms [56,57]. Consequently, we suggest that future studies evaluate how catecholamines influence the metabolic consequences of the social environment, such as those observed here.

In conclusion, metabolic responses of subordinate *N. pulcher* following a stressor were impaired in the presence of a dominant companion, contrary to what would be observed if social buffering had occurred. Instead, our findings suggest that socially induced stress from interactions with a dominant may impose metabolic disadvantages for subordinates, even in social groups with strong prosocial relationships. These findings suggest that there may be social status-specific strategies for energy regulation within dominance hierarchies.

## Data Availability

All data and R scripts supporting this study are publicly available from the Dryad Digital Repository [58]. Supplementary material is available online [59].
